# LDL-cholesterol signaling induces breast cancer proliferation and invasion

**DOI:** 10.1186/1476-511X-13-16

**Published:** 2014-01-15

**Authors:** Catarina Rodrigues dos Santos, Germana Domingues, Inês Matias, João Matos, Isabel Fonseca, José Mendes de Almeida, Sérgio Dias

**Affiliations:** 1 Gulbenkian Programme for Advanced Medical Education, Lisbon, Portugal; 2 Department of Surgical Oncology, Instituto Português de Oncologia de Lisboa, Francisco Gentil, Lisbon, Portugal; 3 Instituto Medicina Molecular, Lisbon, Portugal; 4 Department of Pathology, Instituto Português de Oncologia de Lisboa, Francisco Gentil, Lisbon, Portugal; 5 Faculdade de Medicina Lisboa, Lisboa, Portugal

**Keywords:** LDL-cholesterol, Breast cancer, Tumor growth

## Abstract

Lipids and cholesterol in particular, have long been associated with breast cancer (BC) onset and progression. However, the causative effects of elevated lipid levels and breast cancer remain largely undisclosed and were the subject of the present study.

We took advantage of well-established *in vitro* and *in vivo* models of cholesterol enrichment to exploit the mechanism involved in LDL-cholesterol favouring BC growth and invasiveness. We analyzed its effects in models that mimic different BC subtypes and stages.

Our data show that LDL-cholesterol (but not HDL-cholesterol) promotes BC cells proliferation, migration and loss of adhesion, hallmarks of the epithelial to mesenchymal transition. In vivo studies modeling cholesterol levels showed that breast tumors are consistently larger and more proliferative in hypercholesterolemic mice, which also have more frequently lung metastases*.* Microarray analysis revealed an over expression of intermediates of Akt and ERK pathways suggesting a survival response induced by LDL, confirmed by WB analyses. Gene expression analysis also evidenced an activation of ErbB2 signaling pathway and decreased expression of adhesion molecules (cadherin-related family member3, CD226, Claudin 7 and Ocludin) in the cells exposed to LDL.

Together, the present work shows novel mechanistic evidence that high LDL-cholesterol levels promote BC progression. These data provide rationale for the clinical control of cholesterol levels in BC patients.

## Introduction

Cholesterol is an essential structural component of the cell membrane [1].

Proliferating cells, such as cancer cells, are believed to have increased requirements for cholesterol. To overcome its needs, tumor cells can increase lipid biosynthesis [2], but can also uptake cholesterol from the bloodstream [3,4]. Abnormal cholesterol accumulation is a characteristic of some malignancies [5,6] and the inhibition of cholesterol storage machinery, in breast cancer cell lines was associated with reduced proliferation [7].

The capacity of cancer cell to use exogenous lipids has been pointed up to explain the link between dyslipidemia and high fat diets with cancer [3,7].

Epidemiological studies tried to demonstrate a causal relationship between dyslipidemia and cancer but they have failed [8-12], or found just weak associations [13,14]. However, it should be noted that several of those studies did not account for the lipoprotein fractions, lipid lowering drugs use or the different tumor types, which could all influence the results and their interpretation. Moreover, most studies to date have sought to find a causal link between cancer incidence and lipid levels; significantly less studies tried to explore a possible link in cancer aggressiveness or progression. Thus, for now, the importance of plasma cholesterol in cancer progression remains poorly understood and was the subject of the present study.

We asked whether exposure to a host LDL-cholesterol enriched systemic environment promotes breast cancer progression by activating key signaling pathways and modulating cell behavior. To test this hypothesis we used controlled experimental environments, employing well established *in vitro* and *in vivo* models.

## Methods

### Laboratory methods

#### Cell lines and reagents

The human breast cancer cell line HTB20 and the mouse breast cancer cell line 4 T1 were purchased from the American Type Culture Collection. The human breast cancer cell lines HTB126, MDA MB 231 were kindly provided by Instituto Português de Oncologia Porto.

The cell lines were cultured in DMEM (Gibco Invitrogen, Carlsbad, CA, USA) supplemented with 10% heat-inactivated fetal bovine serum (FBS, Gibco Invitrogen, Carlsbad, CA, USA) and 1% penicillin-streptomycin (Invitrogen life Technologies). Fetal bovine serum lipoprotein free (FBSLF) was purchased from Sigma-Aldrich (Germany) and human plasma low density (LDL) and high density (HDL) lipoproteins were obtained from Calbiochem (Gibbstown, NJ, USA). Mitomycin C was from Sigma-Aldrich (Germany) and Trypsin from (Gibco Invitrogen, Carlsbad, CA, USA).

#### Cell proliferation assay

MDA MB 231, HTB 126, HTB 20 cells (1 × 10^5^/mL) were seeded into 24-well plate in 250 μl DMEM, 10%FBS. After overnight incubation, the medium was replaced by DMEM, 1%FBSLF, for 24 h. Then, the medium was aspirated and cells were incubated with medium containing HDL (100 μg/mL) or LDL (100 μg/mL), at 37°C in 5%CO_2,_ for 24 h or 48 h. The number of living cells was determined by hemocytometer counts (at least 4 counts/well, in quadruplicates), after Trypan Blue test exclusion. The number of cells is expressed as fold change over the control.

#### Migration assay

MDA MB 231 cells were seeded on 24-well plate and grown to confluence in DMEM, 10%FBS. Upon reaching confluence, the medium was replaced by DMEM, 1%FBSLF, for 24 h. Two-hundred-microliter tips were used to make a denuded area (“wound”) in the center of the well. Each well was washed with PBS and treated with HDL (100 μg/mL) or LDL (100 μg/mL) for 24 h. Mitomycin C (0,5 μmol/L, from Sigma) was added to the medium to block cell proliferation. Serial photographs were taken at 0 h, 12 h and 24 h, and cell migration distance was determined by subtracting the values obtained at 0 h from 24 h (at least 4 measurements/well, in quadruplicates). The migration distances are expressed as percentage of the wound closure.

#### Adhesion assay

MDA MB 231 cells (1 × 10^5^/mL) were seeded into 24-well plate in 250 μl DMEM, 10%FBS, overnight, and then replaced by DMEM, 1%FBSLF, for 24 h. After 24 h, cells were left untreated or exposed to LDL (100 μg/mL),overnight. Than wells were washed with PBS, and cells removed with trypsin and reseeded into 24-well plates in 250 μl DMEM, 10% FBS, as defined earlier. After 4 h, cells were washed with PBS and adherent counted. The cells in the supernatant were also counted. The results are shown as the number of adherent or supernatant cells/mL.

#### RNA extraction and microarray analysis

Total RNA was extracted by Trizol method from untreated or LDL treated breast cancer cells (MDA MB 231) and used to study changes in gene expression. The samples were hybridized on an Affymetrix GeneChips at Instituto Gulbenkian de Ciência core facility. The gene expression results were analyzed using Chipster 2.2.0 software. A cutoff of 1.5 fold above or below the house keeping gene expression levels was considered significant. IPA Ingenuity Systems (Ingenuity Systems, Mountain View, CA) was used to exploratory analysis of interactive networks and relevant biological interactions.

#### Protein extraction and Western Blot analysis

Cells were lysed in 50 mM tris, 5 mM EDTA, 2% SDS, pH6.8 buffer containing protease inhibitor cocktail. Lysates were diluted 1:1 in loading buffer (tris–glycerol, 2% SDS, 4% b-mercaptoethanol, 100 mM DTT) and 300 μg proteins were loaded on 10% tris–glycine gels. Proteins were transferred to 0.2 lM nitrocellulose membranes (Hybond-C Extra, GE Healthcare Life Sciences, Roosendaal, Netherlands) and subjected to standard immunoblotting with the antibodies: ERK, pERK, akt, pakt, pJNK, β-actin (all from Cell Signaling, Thecnology). Bands were detected with anti-species HRP conjugate. ImageJ software was used to quantify the density of the bands [15].

#### Statistical analysis

All results, unless otherwise indicated, are expressed as the mean ± standard error of, at least, triplicates. Data were analyzed using unpaired two-tailed Student's t test. *P* values of <0.05 were considered statistically significant.

### *In vivo* models

All animal experiments were performed after approval from Ethics Committee of the Instituto Gulbenkian de Ciência. Animals were housed and maintained in a barrier facility at Instituto Gulbenkian de Ciência. In each experiment, 4–6 week old female mice were injected with breast cancer cells in the right axillary mammary fat pad. Than the test group was subjected to a high cholesterol diet (HD,10%fat, 1,25%cholesterol, 0,5%Na cholate diet, Ssniff, Germany) and the control group fed the standard (normal) mouse diet (ND), with no differences in energy up take values. Food and water were given *ad libitum*. Elevated cholesterol levels were confirmed by standard dosing methods at Clinical Pathology Laboratory of the Instituto Português de Oncologia de Lisboa and parallel groups were used to control the diet effect on lipid profile. In order to test different tumor types and different host backgrounds the following trials were performed:

1) BALB SCID/MDA MB 231 (2 × 10^6^ cells, 4–6 week old female, n = 8), 10 weeks;

2) BALB SCID/HTB 20 (2 × 10^6^ cells, 4–6 week old female, n = 4), 20 weeks;

3) NOD SCID/4 T1 (1 × 10^6^ cells, 4–6 week old female, n = 4), 20 days;

4) BALB C/4 T1 (1 × 10^6^ cells, 4–6 week old female, n = 5), 20 days.

The animals were sacrificed at different times following tumor inoculation, as referred above; mammary tumors, lungs and liver were excised. Tumors were split into two parts, one frozen in liquid nitrogen and stored at -80°C and the other, and other organs, were fixed in 10% neutral buffered formalin. Photos of the tumor were taken and the large diameter measured. Blood was collected, by cardiac puncture, and serum used to determine lipid profile (total cholesterol (TC), LDL, HDL and triglycerides). Tumor sizes (large diameter, mm) are presented as fold change over the control group. Systemic metastases were searched macroscopically during organs collection and microscopically in the lungs and liver.

Immunohistochemical staining for Ki 67 was performed in a Dako Autostainer® (Dako, Glostrup, Denmark) using standard protocols, followed by counting positive cells in an automated cellular imaging system (ACIS® II, Dako, Glostrup, Denmark I), at, Department of Pathology at Instituto Português Oncologia de Lisboa.

An additional trial was done, using statins, in order to test the effect of the reduction of the systemic cholesterol on the tumor. Using as mice model NOD SCID /4 T1, the test group was subjected to a high cholesterol diet (as described above) and treated with simvastatin (5 mg/Kg in 200 μL PBS, 3 days a week, (by gastric tube). Control groups were fed with standard (normal) mouse diet and high cholesterol diet and treated with placebo (200 μL PBS, 3 days a week, (by gastric tube). The mice were injected with tumor cells 4 weeks after starting treatment and treated during 4 weeks more. Animals were sacrificed as described before.

### Statistical analysis

Values are given as the mean ± standard error. Comparisons between control and test mouse samples were performed using the unpaired two-tailed Student´s t test. The number of mice used for each experiment is indicated in the Figure. *P* values of <0.05 were considered statistically significant.

## Results

### LDL-cholesterol stimulation induces breast cancer cell lines proliferation, migration and reduces cell adhesion

**Figure 1:**
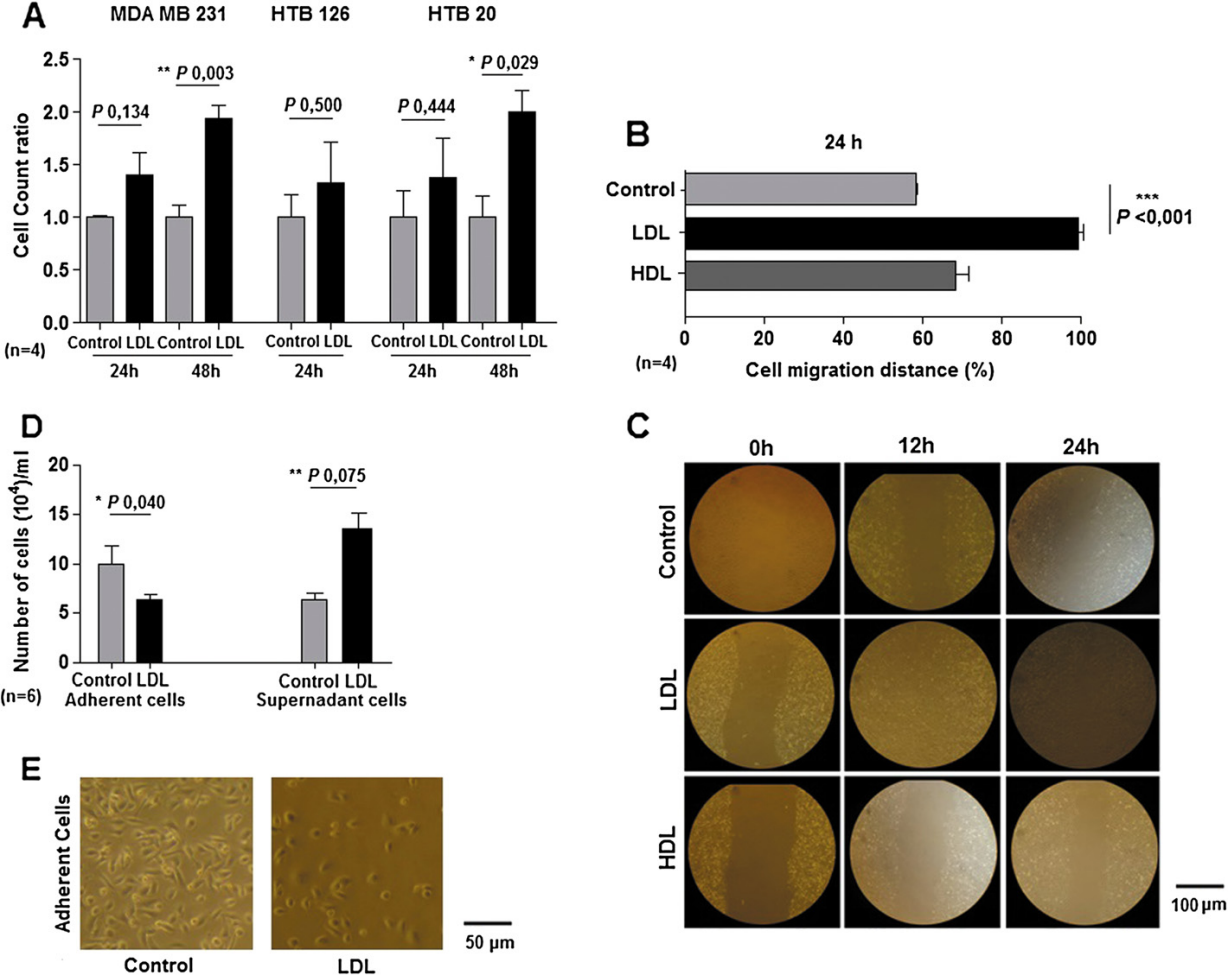
**Proliferation, migration and loss of adhesion induced by LDL in in breast cancer cell lines. A**, Number of cells (MDA MB 231, HTB 126, HTB 20) after 24 h and 48 h of LDL (100 μg/mg) exposure is consistently higher than control (non-treated) condition. **B** and **C**, Cells (MDA MB 231) were wounded and then cultured for 24 h, with LDL (100 μg/mL), HDL (100 μg/mL) or control conditions. Cell migration into the wound was examined by phase-contrast microscopy and migration distance is indicated as the percentage of the wound closure at 24 h. Representative photos are shown (original magnification 100x C). **D** and **E**, Number of cells (MDA MB 231) adherent and no adherent cells, at 4 h after being removed and reseeded on its primary conditions (control and LDL100 μg/mL) shows that LDL treated cells lose matrix adhesion compared to control. Representative photos are shown (original magnification 200x E). *P* value and the number of the experiments are represented in the figure. Columns mean; bars ± SEM.

We examined the effect of LDL on breast cancer cells proliferation and found that the number of viable cells increased after LDL-cholesterol stimulation in all cell lines, reaching statistical significance in MDA MB 231 and HTB 20 at 48 h (Figure 1A). Further dissection of LDL induced phenotype was performed on MDA MB 231 cells. In detail, we also determined whether LDL could induce the migration of breast cancer cells in “wound/starch assays”, in the presence of mitomycin C to inhibit cell proliferation, and we found that LDL induced migration of MDA MB 231 cells, promoting *in vitro* wound closure. Importantly, HDL was used as control and did not promote cell migration (Figure 1B and C).

Thereafter we tested if cells incubated with LDL changed their adhesive behavior. As shown in Figure 1D and E, LDL pre-treated MDA MB 231 cells, lost their adhesion to the matrix compared to control (untreated) conditions.

Together, these data suggest that LDL exposure of breast cancer cells affects their adhesive properties, favoring cell migration and proliferation.

### LDL-cholesterol induced gene expression changes in breast cancer cell lines

**Figure 2:**
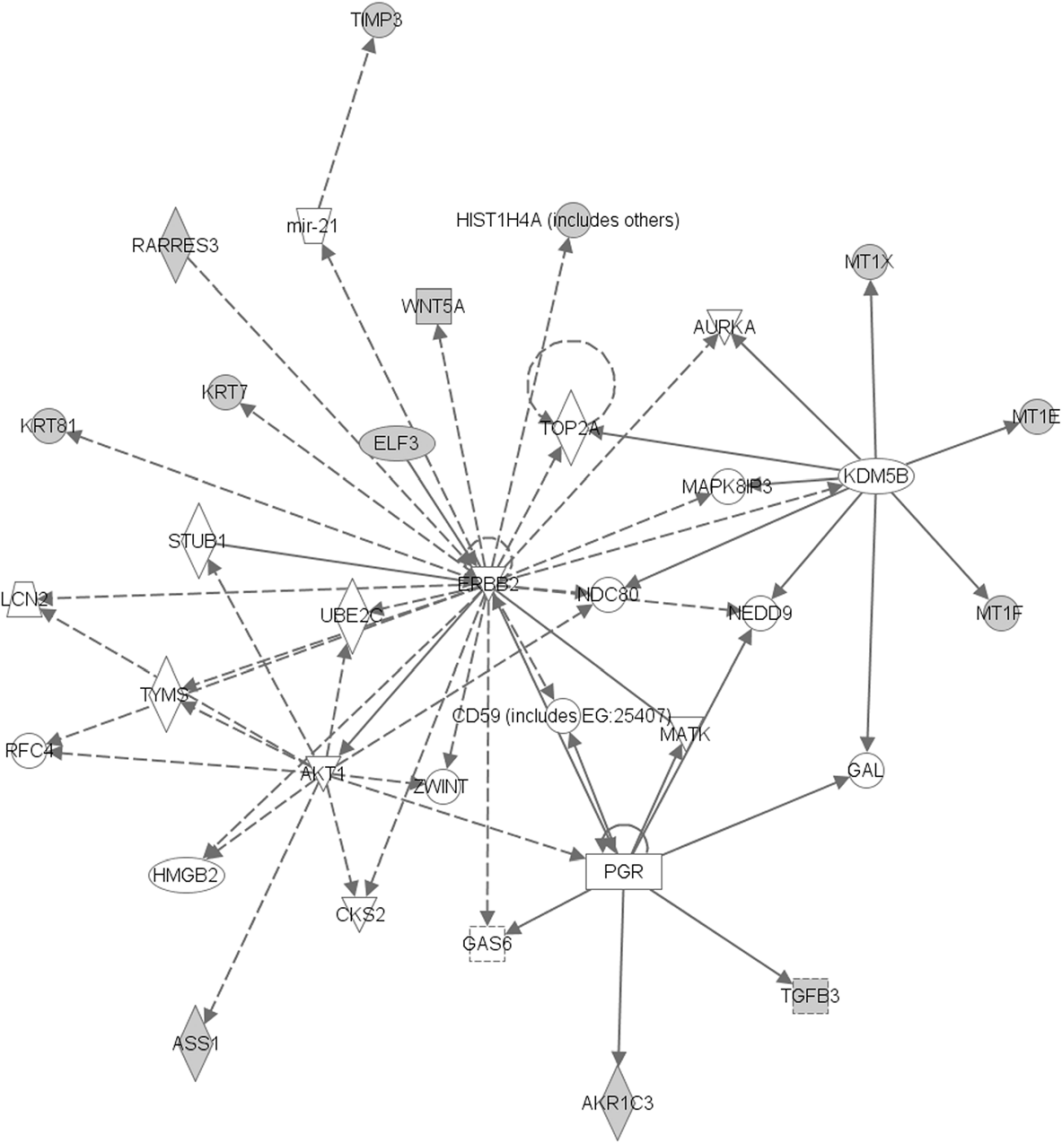
**Activated cellular networks, at 48 h, in LDL treated breast cancer cell line.** Gene expression analysis of breast cancer cells MDA MB231 exposed to LDL (100 μg/mL), for 48 h shows up regulation of molecules involved in activation of ERK, akt and ErbB2 pathways. Grey nodes are genes overexpressed, white nodes are predicted genes. Smooth lines represent direct interactions. Dashed lines represent indirect interactions.

Having shown LDL induced phenotypic changes on breast cancer cells, next we sought to demonstrate a causal mechanistic link between these changes and the activation of signaling intermediates and pathways that could explain the altered properties. For this purpose we performed Affymetrix microarray analysis of untreated versus LDL treated breast cancer cells. We found an over expression (fold change ≥1,5) of 147mapped genes and down regulation of 95 mapped genes, at 48 h. The great majority of these genes were related to cell survival and proliferation pathways (Additional file 1: Table S1). Among altered expression genes are also, the down regulation of the adhesion molecules cadherin-related family member3 (-1,53 fold change), CD226 (-1,52 fold change), Claudin7 (-1,52 fold change), Ocludin (-1,54 fold change) and integrinβ8 (-1,49 fold change). Exploratory analysis of the significant gene interactions, found an activation of akt, ERK and JNK networks, all in the dependency of the ErbB2 pathway (Figure 2).

**Figure 3:**
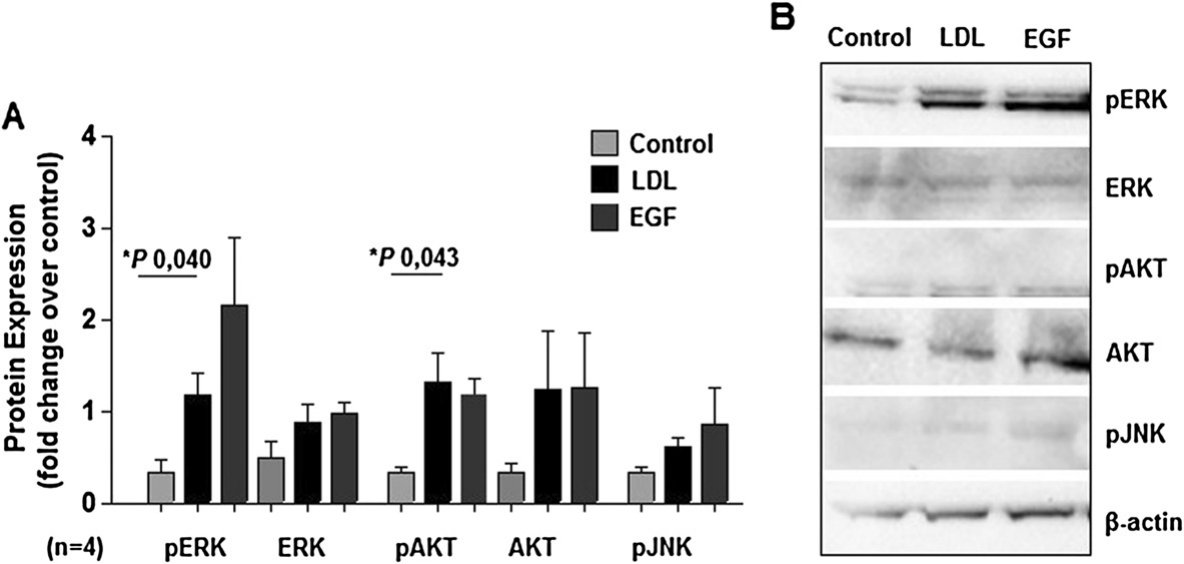
**LDL induces ERK and akt protein phosphorylation. A**. Cells (MDA MB 231) exposed to LDL show higher expression of phosphorylated ERK and akt, without significant increase in respective total protein. Phosphorilation of JNK is also higher, without reaching statistical significance. Epidermal growth factor (EGF) stimulation was used as positive control and induced, as expected, increase ERK, akt and JNK phosphorylation. **B**. Representative photos of western blot membranes are shown. *P* value and the number of the experiments are represented in the figure. Columns mean; bars ± SEM.

By western blotting analysis we confirmed the raised phosphorylation of akt and ERK, but not of JNK (Figure 3).

These data evidence the LDL effect on the breast cancer cell promoting survival, proliferation and migration.

### High LDL-cholesterol promotes breast cancer growth in animal models

**Figure 4:**
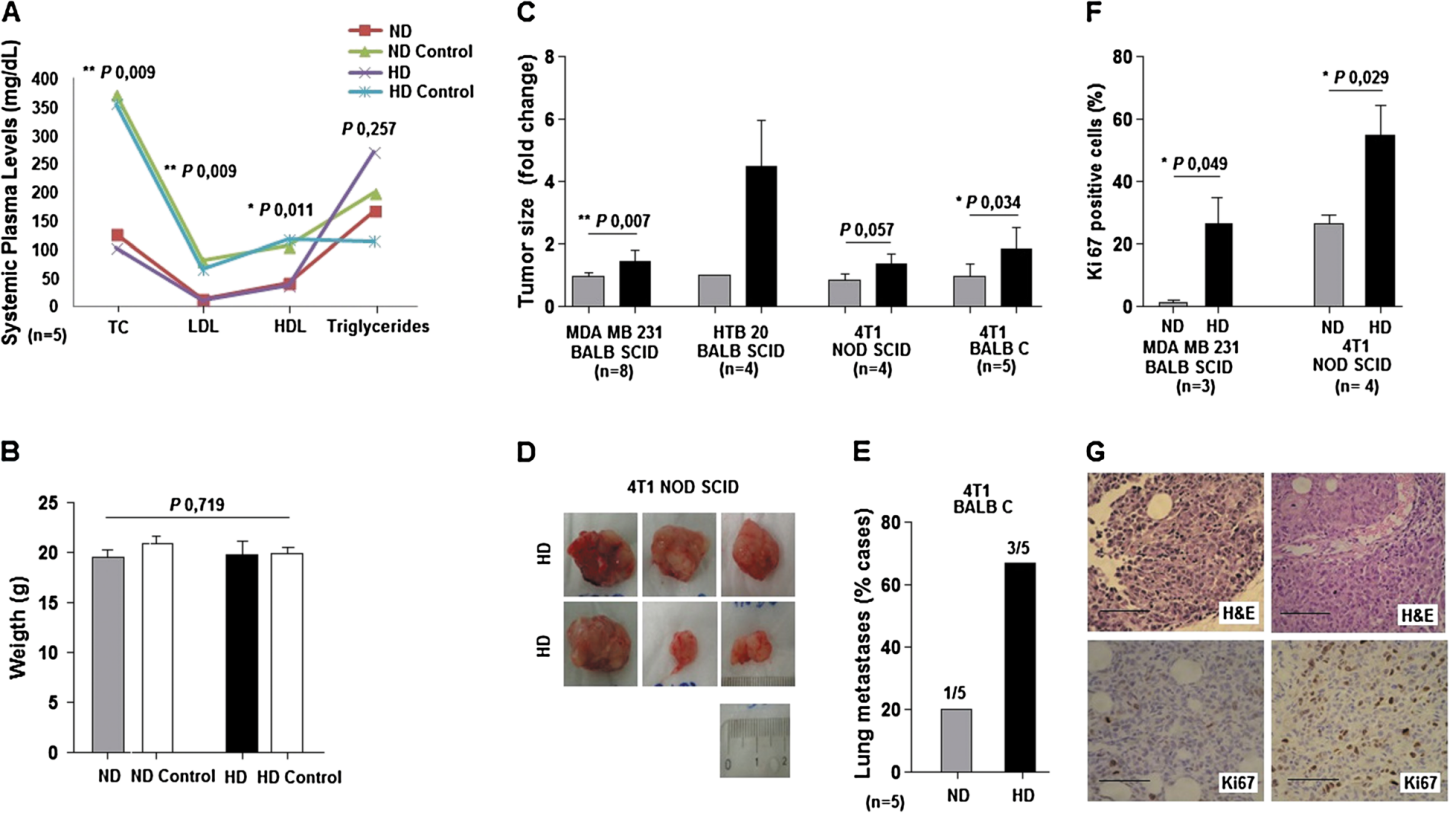
**Hypercholesterolemic diet induces a breast cancer phenotype characterized by large and more proliferative tumors. A** and **B**. HD fed mice have raised levels of total cholesterol (TC), low density lipoprotein (LDL) and high density lipoprotein (HDL). There are no statistical significant differences in triglycerides level or animal weight, as exemplified to MDA MB 231/BALB SCID trial. (Results of the other experiments are in supplementary data.) Animal in the same diet but without tumor cells inoculation were used as control to lipid profile parameters. **C** and **D**. HD fed mice show large tumors when compared to ND fed mice. The mammary tumor large diameter was measured (as exemplified to 4 T1 /NOD experiment B) and the differences are shown as the fold change over the ND fed mice. **E**. HD fed mice are more likely to have lung metastasis (100%) than ND fed mice (33%). **F** and **G**. HD fed mice have more proliferative tumors as confirmed by Ki67 immuno staining. *P* value and the number of the experiments are represented in the figure. Columns mean; bars ± SEM. Scale bar 100 μm.

HD fed mice showed high levels of TC, LDL and HDL (Figure 4A). However, there were no statistically significant differences in triglycerides levels or animal weight (Figure 4A and B). The values of the mouse lipid profile in each experiment are shown in Additional file 1: Table S2. These data validate this high fat diet model as a good model to specifically address the effects of elevated cholesterol levels.

HD promoted breast tumor growth in all models tested, with statistically significant differences in BALB SCID inoculated with MDA MB 231 and BALB C inoculated with 4 T1 cells (Figure 4C). Tumors of HD fed mice showed a proliferative ratio that was 20% higher than tumors from ND fed mice, assessed by immunohystochemistry (for Ki67 positive cells) (Figure 4F and G).

Lung metastases were observed macroscopically and microscopically in the NOD SCID and BALB C/4 T1 models. No significant differences were registered the first model, but BALB C/4 T1 hypercholesterolemic mice showed a higher frequency of lung metastasis, at the end of the trial, compared to ND fed mice (Figure 4E). No liver metastases were registered. Two mice of the longest BALB SCID/HTB 20 trial died, one of each group. No other mice deaths occurred.

### Statins treatment does not reduced systemic LDL level in mice trial

The treatment with statins did not reduce cholesterol levels of HD fed mice (Additional file 2: Figure S1A). Tumor size of HD fed mice treated with statins show no significant difference from HD fed control mice, in this trial (*P* 0,104) (Additional file 2: Figure S1 B and C).

## Discussion

Obesity and dyslipidemia, have long been linked with a possible increase in the likelihood of developing cancer. This possible causal relationship has gained momentum in the light of the observed obesity “epidemic” and the recognized increased incidence of cancer in western countries [16]. Moreover, the same trend in obesity, dyslipidemia and cancer incidence has been seen in Asian with the growing incorporation of western lifestyles [17,18].

However, few studies have tried to find causal and mechanistic associations between increased lipid levels and- not cancer incidence- but cancer behavior. This was tested in the present study, using well established *in vitro* and *in vivo* models of hypercholesterolemia and breast cancer.

In breast cancer cell lines, representative of different tumor subtypes and stages, we found that LDL (but not HDL) exposure induces cell proliferation, migration and loss of adhesion, hallmarks of the process of epithelial to mesenchymal transition [19].

Others have demonstrated that in some breast cancer cell lines, HDL induces proliferation [20,21]. We saw a discrete effect of HDL in ER negative cells proliferation (data not shown, not significant), but no influence in migration or adhesion properties.

Previous studies, also showed that LDL induces proliferation [7,20] and migration [4] of ER negative, but not ER positive breast cancer cell lines. We used an ER positive breast cancer cell line HTB 20 (BT 474) and LDL induced similar phenotype changes. HTB 20 breast cancer cells usually express ErbB2 receptor [22].

Our own unpublished data shows that there is an association between high plasma LDL level and ErbB2 positive breast cancer [23]. Also the exploratory analysis of gene expression microarrays suggested an upstream activation of an epidermal growth factor receptor (EGFR).

Human epidermal growth factor receptor -2 (Her2-neu/ ErbB2) is a membrane tyrosine kinase and oncogene that is overexpressed and gene amplified in about 20% of breast cancers [24].

Independent investigations have demonstrate that cell cholesterol depletion using Methyl-N-Cyclodextrin, reduces fluidity of the membrane and enhances phosphorilation and consequent activation of EGFR downstream cascade [25-28]. Orr et al [27] specifically demonstrated this effect in ErbB2 receptor. Since EGFR could be activated in a ligand-independent manner [28], we suggest that cholesterol mobilization across the cell membrane may be the responsible for changes in membrane equilibrium/ disorganization leading to ErbB2 activation, rather than the absolute cholesterol content itself. But, as mentioned earlier, this hypothesis remains to be fully exploited in future studies.

Gene and protein expression analysis of breast cancer cells stimulated with LDL revealed that the proliferative effect induced by LDL may be dependent on Akt and ERK pathways activation. Gene expression analysis also suggested decreased expression of adhesion molecules such as cadherin-related family member 3, CD226, Claudin 7 and Ocludin, upon breast cancer cells exposure to LDL. These findings could explain the loss of adhesion and increased migration in the functional tests, when cells were exposed to LDL and represent hallmarks of the epithelial to mesenchymal transition*,* characteristic of tumor progression.

To systemically test the *in vitro* results, hypercholesterolemia, controlling obesity, was induced in mice of different background (including NOD SCID mice, which are non-obese mice) with different cells lines (to mimic different tumor subtypes) and data consistently showed greater tumor growth in the high LDL groups. This is in accordance with the results reported by Llaverias et al, in breast and prostate genetic mice models [29,30]. Our xenograft models have the advantage of being more representative of breast cancer heterogeneity.

Tumors grown in hypercholesterolemic mice were characterized by higher proliferative ratios, measured by Ki67 immunostaining, which is considered a worse prognostic marker [31]. Higher frequency of lung metastasis was also observed in HD fed mice inoculated with the 4 T1 xenogeneic breast cancer cell line. In models using human breast cancer cell lines we were not able to detect systemic metastasis, either macro or microscopically, may be because those cells do not induce metastasis in mice (reports are very scarce) or because the length of our experiments was not sufficient.

We tried to reverse the hypercholesterolemic-induced phenotype by treating mice with lipid lowering drugs. However we did not achieve systemic LDL levels reduction with statins, in our model. This is a limitation of our work but such difficulty was also found by others [32]. Rats and mice that are commonly used in experimental cancer studies are generally unresponsive to the hypocholesterolemic effects of statins [32].

In humans, the effect of statins in cancer prevention and treatment remains controversial. Two large meta-analyses from 2006 described a neutral effect of satins in cancer incidence [33,34]. A very recent cohort study crossing statins prescription/pharmacy dispense and cancer related-mortality in Denmark National databases [35], proved that statins use before diagnosis of cancer reduces cancer-related mortality. Although some limitations are found and pointed by the authors and others [36], such as lack of follow up and co-morbilities information, the association seem to be plausible, in the study population, suggesting a role for primary prevention.

While assuming that a putative effect of statins is due to plasma cholesterol levels reduction, the action of statins in cancer cells is not well known. Statins decrease intracellular cholesterol synthesis by targeting hidroxi-3-methyl-glutaril-CoA redutase 3. This may lower the products of mevalonate pathway involved in cell proliferation and by this mechanism prevent tumor progression. Some of these effects have been demonstrated in *in vitro* experiments [37]. However, there is poor evidence of direct effect of statins in cancer cells when taken orally [38]. Therefore, much remains to be learned and developed with regards to lipid control in breast cancer setting.

**Figure 5:**
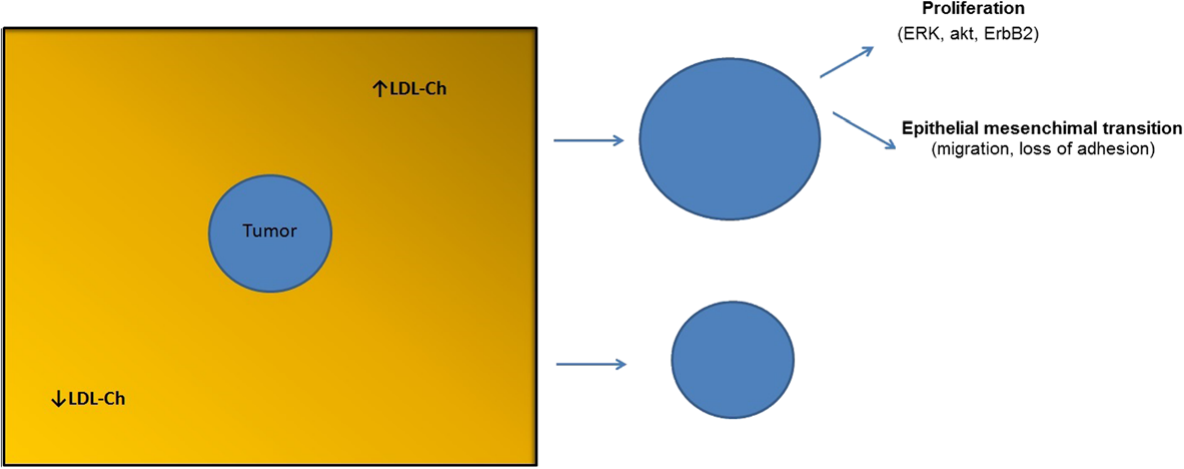
**Phenotype of breast tumors exposed to high levels of cholesterol-Proposed Model.** Breast tumors exposed to high levels of LDL-cholesterol are larger and show increased proliferation and migration while losing epithelial adhesion properties.

Taken together, our findings show that breast cancer exposed to an LDL-rich host macro environment may be in survival advantage, which will ultimately result in a more aggressive breast cancer phenotype (Figure 5). Our results are supported by functional studies in cell lines and animal models of breast cancer and are in strong accordance with clinical data.

The study exposes the importance of controlling systemic cholesterol levels in breast cancer prevention and treatment and reveals LDL as a biomarker of aggressiveness.

## Competing interests

The authors declare that they have no competing interests.

## Authors*'* contributions

CRS designed the study, performed the functional studies, developed the breast cancer mice models, collected and analyzed data and wrote the manuscript. GD Breast cancer mice models, performed the protein extraction and Western Blot analysis, collected and analyzed data. IM cultured and expanded breast cancer cells lines, performed the RNA extraction and microarray analysis. JM performed the immunohistochemical studies. IF analyzed and validated the pathological data, revised the manuscript. JMA analyzed and validated data, revised the manuscript. SD designed and coordinated the study, analyzed data and wrote the manuscript. All authors read and approved the final manuscript.

## Supplementary Material

Additional file 1: Table S1 Gene expression of LDL treated vs Control MDA MB 231. Table S2 . Lipid Profile in mice trails. Click here for file

Additional file 2: Figure S1 Tumor size of hypercholesterolemic diet fed mice treated with statins show no significant differences from hypercholesterolemic diet fed control mice. **A** HD fed mice have raised levels of total cholesterol (TC) and low density lipoprotein (LDL) and no significant differences in high density lipoprotein (HDL) and triglycerides levels compared ND. Treatment with statins 5 mg/dL, 8 weeks, does not change lipid profile in the HD fed mice. **B** and **C** NOD SICD/ 4 T1 mice model fed with HD show no significant differences in tumor size compared to HD fed mice treated with statins 5 mg/mL. *P* value and the number of the experiments are represented in the figure. Columns mean; bars ± SEM. Click here for file
