# Supplementary material for: LDL-cholesterol signaling induces breast cancer proliferation and invasion
Source: Lipids Health Dis. 2014 Jan 15;13:16. doi: 10.1186/1476-511X-13-16 (PMC3896822; doi:10.1186/1476-511X-13-16)
Supplement: Additional file 1: Table S1 — Gene expression of LDL treated vs Control MDA MB 231. Table S2 . Lipid Profile in mice trails. [file 1476-511X-13-16-S1.docx]

| **Table S1: Gene expression of LDL treated vs Control MDA MB 231** | | | |
| --- | --- | --- | --- |
| **Molecules in Network** | **Score** | **Focus Molecules** | **Top Functions** |
| Up regulated 6h |  |  |  |
| ANGPTL4,FFAR3,GPR152,  KIR2DL3,MAL2,MTRF1L,POLR3G,  RN7SK,ROPN1,SLC25A20,SPRR2G | 28 | 12 | Organismal Development, Lipid Metabolism |
| CISH,CPT1A,IL24,PDGFD,  PRLR,RETNLB,TNIP3 | 25 | 11 | Cancer, Reproductive System Disease, Cell Morphology |
| TMEM229A | 3 | 1 | Embryonic Development, Organ Development, Organismal Development |
| LGALS9C | 3 | 1 | Digestive System Development and Function, Embryonic Development, Tissue Morphology |
| GRAMD1B,MAPK1 | 3 | 1 | Organ Morphology, Nucleic Acid Metabolism |
| PCDHA8 | 3 | 1 | Embryonic Development, Organ Development, Organismal Development |
| LCE2C | 3 | 1 | Cell Death and Survival, Cellular Assembly and Organization, Cellular Development |
| PCDHA13 | 3 | 1 | Cell Cycle, Embryonic Development, Renal and Urological System Development and Function |
| GGT2 | 3 | 1 | Drug Metabolism, Protein Synthesis |
| S100Z | 2 | 1 | Cancer, Carbohydrate Metabolism, Cardiovascular System Development and Function |
| XCL2 | 2 | 1 | Reproductive System Disease, Cellular Movement, Cell Signaling |
| Down regulated 6h |  |  |  |
| BHLHE40,BTG1,CCNG2,FASN,  HMGCR,IDI1,INSIG1,KRT80,  LIFR,MVK | 17 | 11 | Cellular Growth and Proliferation, Cellular Development, Cell Cycle |
| CLDN7 | 2 | 1 | Cancer, Cell Cycle, Cell Morphology |
| TP53INP1 | 2 | 1 | Endocrine System Disorders, Gastrointestinal Disease, Inflammatory Disease |
| AIM2 | 2 | 1 | Cell-To-Cell Signaling and Interaction, Inflammatory Response, Gastrointestinal Disease |
| NPY1R | 2 | 1 | Energy Production, Molecular Transport, Nucleic Acid Metabolism |
| Up regulated 48h |  |  |  |
| AKR1C3,ASS1,ELF3,HIST1H4A,  KRT7,KRT81,MT1E,MT1F,MT1X,  RARRES3,TGFB3,TIMP3,WNT5A | 15 | 13 | Cancer, Gastrointestinal Disease, Cellular Development |
| ALOX5AP,C15orf48,CABLES1,  CD74,CRISPLD2,EPAS1,GPR56,  HLADRA,KIAA1199,PHB2,  S100A2,S100A4,TACSTD2 | 15 | 13 | Cancer, Gastrointestinal Disease, Dermatological Diseases and Conditions |
| HES1,ITGB4,LPCAT3,PBX1,  PTGS1,STAT5B,TNFSF10 | 5 | 6 | Cell Death and Survival, Cellular Development, Cellular Growth and Proliferation |
| ANGPTL2 | 1 | 1 | Connective Tissue Disorders, Developmental Disorder, Skeletal and Muscular Disorders |
| SIX1 | 1 | 1 | Cellular Development, Cellular Growth and Proliferation, Tumor Morphology |
| Down regulated 48h |  |  |  |
| CSF2,DHCR7,DHCR24,EGR1,  HMGCR,IDI1,IL11,  IL24,INSIG1,MVK,KRT15 | 20 | 11 | Cellular Development, Cellular Growth and Proliferation, Lipid Metabolism |

| **Table S2 . Lipid Profile in mice trails** | | | | | |
| --- | --- | --- | --- | --- | --- |
|  | **Normal Diet** | **Hypercholesterolemic Diet** |  | **Normal Diet** | **Hypercholesterolemic Diet** |
|  | **Tumor** | |  | **Control** | |
| ***Mice model/ Weight/Lipid Profile variable*** | **MDA MB231**  **BALB SCID**  **N=8** | **MDA MB231**  **BALB SCID**  **N=8** | ***P value*** | **MDA MB231**  **BALB SCID**  ***N=5*** | **MDA MB231**  **BALB SCID**  ***N=5*** |
| ***Weight (g)*** | 19,6(±1,79) | 19,9(±2,87) | 0,6389 | 20,92(±1,50) | 19,9(±1,85) |
| ***TC (mg/dl)*** | 126,8(±0,73) | 372,3(±107,66) | 0,0095 | 102,1(±17,42) | 356(±84,79) |
| ***LDL (mg/dl)*** | 11,8(±25,19) | 79,8(±17,29) | 0,0095 | 9,7(±4,41) | 66(±23,29) |
| ***HDL (mg/dl)*** | 40,3(±17,44) | 106,8(±55,78) | 0,0114 | 36,8(±8,76) | 117,2(±42,23) |
| ***Triglycerides (mg/dl)*** | 166,8(±47,77) | 200(±34,33) | 0,2571 | 271(±79,92) | 114,7(±26,54) |
|  | **HTB 20**  **BALB SCID**  **N=3** | **HTB 20**  **BALB SCID**  **N=3** | ***P value*** | **HTB 20**  **BALB SCID**  **N=2** | **HTB 20**  **BALB SCID**  **N=2** |
| ***Weight (g)*** | 19,9 (±1,90) | 19,4(±1,82) | 0,700 | 19,6(±1,06) | 17,0(±2,12) |
| ***TC (mg/dl)*** | - | 345,5(±34,05) |  | 125 | 262 |
| ***LDL (mg/dl)*** | 16,5(±14,85) | 82(±3,46) | 0,200 | 13,5(±2,12) | 111(±14,14) |
| ***HDL (mg/dl)*** | - | 120(±11,728) |  | - | - |
| ***Triglycerides (mg/dl)*** | 121 | 123,5(±3,54) |  | 142 | - |
|  | **4T1**  **NOD SCID**  **N= 4** | **4T1**  **NOD SCID**  **N=** | ***P value*** | **4T1**  **NOD SCID**  **N=2** | **4T1**  **NOD SCID**  **N=2** |
| ***Weight (g)*** | 19,1(±2,85) | 20,2(±2,17) | 1,000 | 21,1(±0,40) | 23,3(±0,85) |
| ***TC (mg/dl)*** | 95,67(±22,9) | 278,7(±58,11) | 0,0071 | 199,0(±173,95) | 333,5(±99,70) |
| ***LDL (mg/dl)*** | 17,3(±4,04) | 105,3(±47,16) | 0,0323 | 5(±5,65) | 50,5(±23,33) |
| ***HDL (mg/dl)*** | 23(±7,94) | 53,7(±16,92) | 0,0468 | 48(±43,84) | 54,5(±34,65) |
| ***Triglycerides (mg/dl)*** | 93,7(±49,17) | 157(±58,39) | 0,2240 | 250(±207,89) | 317,5(±135,06) |
| TC: Total cholesterol; LDL: Low Density Lipoprotein; HDL: High Density Lipoprotein. Values are represented by mean±SD.  *P* value : Student t test | | | | | |

**Figure 5**
