# Supplementary figures and images for: LDL-cholesterol signaling induces breast cancer proliferation and invasion
Source: Lipids Health Dis. 2014 Jan 15;13:16. doi: 10.1186/1476-511X-13-16 (PMC3896822; doi:10.1186/1476-511X-13-16)

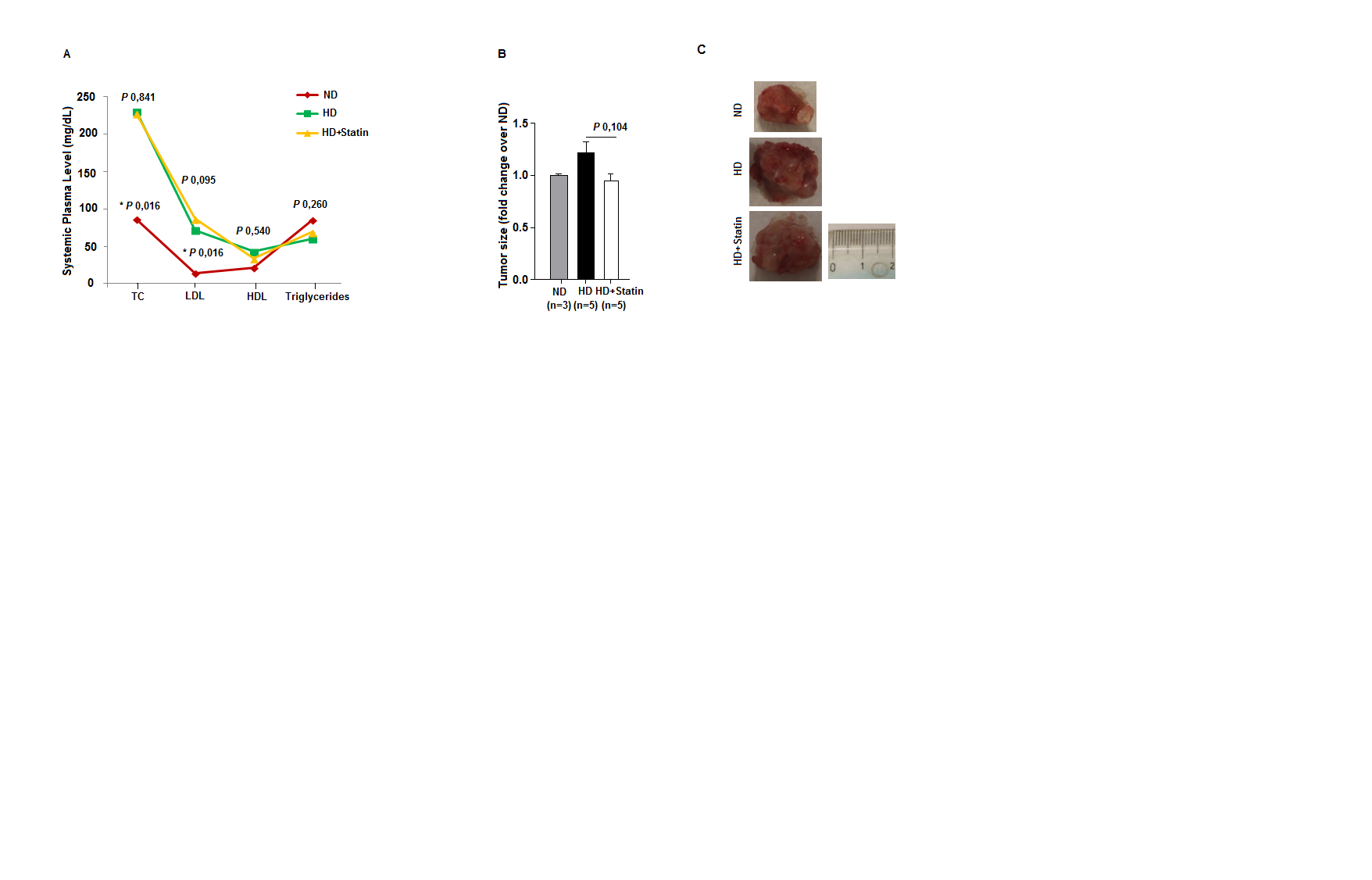

Supplement: Additional file 2: Figure S1 — Tumor size of hypercholesterolemic diet fed mice treated with statins show no significant differences from hypercholesterolemic diet fed control mice. A HD fed mice have raised levels of total cholesterol (TC) and low density lipoprotein (LDL) and no significant differences in high density lipoprotein (HDL) and triglycerides levels compared ND. Treatment with statins 5 mg/dL, 8 weeks, does not change lipid profile in the HD fed mice. B and C NOD SICD/ 4 T1 mice model fed with HD show no significant differences in tumor size compared to HD fed mice treated with statins 5 mg/mL. P value and the number of the experiments are represented in the figure. Columns mean; bars ± SEM. [file 1476-511X-13-16-S2.png]
